# Evaluating the systemic right ventricle by CMR: the importance of consistent and reproducible delineation of the cavity

**DOI:** 10.1186/1532-429X-10-40

**Published:** 2008-08-19

**Authors:** Michiel M Winter, Flip JP Bernink, Maarten Groenink, Berto J Bouma, Arie PJ van Dijk, Willem A Helbing, Jan GP Tijssen, Barbara JM Mulder

**Affiliations:** 1 Department of Cardiology, Academic Medical Center, Meibergdreef 9, Amsterdam, The Netherlands; 2 Department of Radiology, Academic Medical Center, Meibergdreef 9, Amsterdam, The Netherlands; 3 Department of Cardiology, University Medical Center Nijmegen, Geert Grooteplein-Zuid 10, Nijmegen, The Netherlands; 4 Department of Cardiology, Erasmus MC – Sophia Children's Hospital, Dr. Molewaterplein 60, Rotterdam, The Netherlands; 5 Department of Cardiology, University Medical Center Utrecht, Heidelberglaan 100, Utrecht, The Netherlands

## Abstract

**Background:**

The method used to delineate the boundary of the right ventricle (RV), relative to the trabeculations and papillary muscles in cardiovascular magnetic resonance (CMR) ventricular volume analysis, may matter more when these structures are hypertrophied than in individuals with normal cardiovascular anatomy. This study aimed to compare two methods of cavity delineation in patients with systemic RV.

**Methods:**

Twenty-nine patients (mean age 34.7 ± 12.4 years) with a systemic RV (12 with congenitally corrected transposition of the great arteries (ccTGA) and 17 with atrially switched (TGA) underwent CMR. We compared measurements of systemic RV volumes and function using two analysis protocols. The RV trabeculations and papillary muscles were either included in the calculated blood volume, the boundary drawn immediately within the apparently compacted myocardial layer, or they were manually outlined and excluded. RV stroke volume (SV) calculated using each method was compared with corresponding left ventricular (LV) SV. Additionally, we compared the differences in analysis time, and in intra- and inter-observer variability between the two methods. Paired samples t-test was used to test for differences in volumes, function and analysis time between the two methods. Differences in intra- and inter-observer reproducibility were tested using an extension of the Bland-Altman method.

**Results:**

The inclusion of trabeculations and papillary muscles in the ventricular volume resulted in higher values for systemic RV end diastolic volume (mean difference 28.7 ± 10.6 ml, p < 0.001) and for end systolic volume (mean difference 31.0 ± 11.5 ml, p < 0.001). Values for ejection fraction were significantly lower (mean difference -7.4 ± 3.9%, p < 0.001) if structures were included. LV SV did not differ significantly from RV SV for both analysis methods (p = NS). Including structures resulted in shorter analysis time (p < 0.001), and showed better inter-observer reproducibility for ejection fraction (p < 0.01).

**Conclusion:**

The choice of method for systemic RV cavity delineation significantly affected volume measurements, given the CMR acquisition and analysis systems used. We recommend delineation outside the trabeculations for routine clinical measurements of systemic RV volumes as this approach took less time and gave more reproducible measurements.

## Background

The number of adult patients with a congenital heart defect is steadily increasing with the constant improvement of cardiac surgery. A substantial portion of these patients has a morphologic right ventricle (RV) supporting the systemic circulation (e.g. patients with a congenitally corrected transposition of the great arteries (ccTGA) or a complete transposition of the great arteries (TGA) after an atrial switch operation). Medium term survival in patients with a systemic RV is relatively good. However, long-term outcome is unknown, and morbidity is worrisome, with RV dysfunction, tricuspid valve regurgitation, and arrhythmias being the main constituents. [1-4]

Assessment of ventricular function is of particular importance, as European guidelines consider it an important part of preoperative assessment, (peri-operative) management and follow-up of patients with any congenital heart defect. [5] Moreover, decisions on the timing of surgical intervention in patients with a systemic RV are frequently based on RV function. [6,7] Therefore, the importance of having an accurate and reproducible diagnostic tool for the evaluation and follow-up of systemic RV volumes and function is evident.

For the assessment of subpulmonary and systemic RV volumes and function Cardiovascular Magnetic Resonance (CMR) is considered the gold standard. [8-15] In individuals with normal cardiac anatomy the influence of trabeculations and papillary muscles on measured ventricular volumes seems of marginal importance. The differences in measured ventricular volumes and function when these structures are included in the ventricular cavity, compared to when structures are excluded, are small and unlikely to influence clinical decision making.[16,17] However, patients with a systemic RV pose a challenge, as the method of delineating the cavity relative to the hypertrophied trabeculations and papillary muscles could affect RV volume and function measurements. [18]

**Figure 1:**
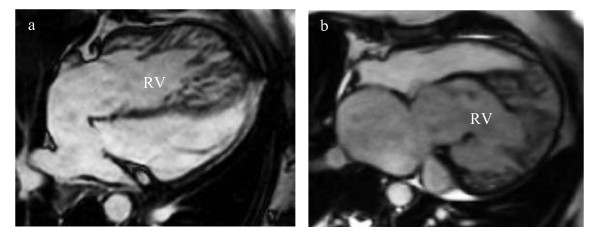
Four chamber image from a multi-phase steady-state free precision sequence of the highly trabeculized systemic RV in a patient with an atrially switched TGA (a) and in a patient with a congenitally corrected TGA (b).

To our knowledge, no study has ever addressed the issue of CMR analysis methods in this patient group. Aim of the present study was to evaluate the impact of trabeculations and papillary muscles on systemic RV measurements, by comparing a CMR analysis method in which trabeculations and papillary muscles were included in the RV volume to an analysis method in which these structures were excluded from RV volume (figure 1). Additionally, differences in analysis time and intra- and inter-observer reproducibility between analysis methods were evaluated.

## Methods

### Study population

A total of 29 adult patients (69% male, mean age 34.7 ± 12.4 years) with a systemic RV underwent CMR for the evaluation of RV volumes and function. Twelve patients had a ccTGA, 17 patients an atrially switched TGA. The Institutional Review Boards of all three participating tertiary referral centers approved the study protocol. Written informed consent was obtained from all patients prior to participation in the study.

### Image acquisition

Image acquisition was performed by CMR, using a 1,5 Tesla scanner (Siemens Avanto, Erlangen, Germany), using standardly available sequences to assess ventricular volumes. After visualizing the long and short axes of the heart, a multi-phase steady-state free precession sequence (SSFP) with retrospective electrocardiographic triggering was applied to visualize two-chamber, three-chamber and four-chamber views. Guided by these views, a multislice and multiphase SSFP sequence was applied perpendicular to the ventricular septum, encompassing the total heart. These sequences were individually adjusted to acquire short axis slices with optimal spatial and temporal resolution. Typical parameters were: flip angle: 50–70 degrees; repetition time: 3–4 msec; echo time: 1–2 msec; temporal resolution: 40 msec, 1–2 × 1–2 mm/pixel in-plane spatial resolution, 8 mm slice thickness, and 1 mm interslice gap. This resulted in 9 to 15 slices to cover the whole heart. CMR images were acquired during repeated end-expiratory breath holds.

### Image analyses

For CMR image analysis two independent observers (MW, FB) used MASS Analytical Software System (Medis, Leiden, the Netherlands). Cine loops were used to choose end diastole (ED) and end systole (ES). ED was defined as the phase with the largest RV (and left ventricular (LV)) area and ES as the phase with the smallest RV (and LV) area. The slices at the base of the heart were considered to be in the ventricle if the blood was at least half surrounded by ventricular myocardium. Cine loop movies in phase and slice were used in case the distinction between the ventricles, atria and great vessels was unclear. Moreover, four-chamber views in phase with the short axis views were available. Tracing was performed manually on each ED and ES short-axis view.

The sums of the traced contours in ED en ES were used to calculate ED volume (EDV) and ES volume (ESV) using a disc summation technique. EDV and ESV were used to calculate Stroke Volume (SV) and Ejection Fraction (EF). SV was defined as EDV - ESV, and EF as [(EDV - ESV)/EDV] × 100%.

### CMR analysis methods

**Figure 2:**
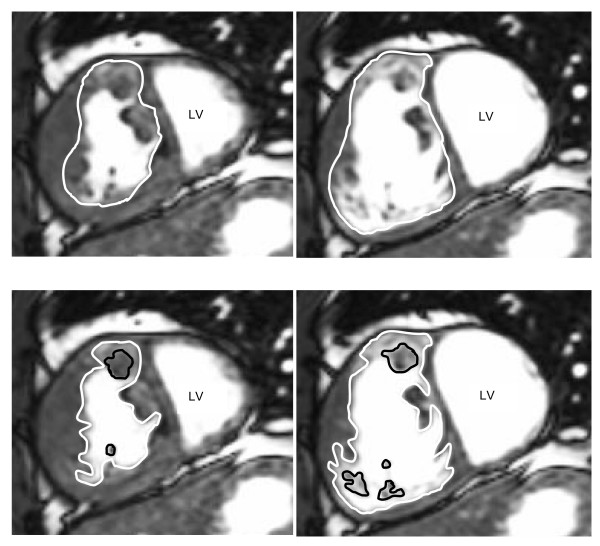
**Assessment of systemic right ventricular volumes using two different analysis protocols**. Short axis view from a multi-phase steady-state free precision sequence in end systole (left) and end diastole (right) obtained in a patient with an atrially switched TGA demonstrating the two analysis protocols. Method **A **depicts the inclusion of trabeculations and papillary muscles in the ventricular cavity. Method **B **depicts the exclusion of trabeculations and papillary muscles from the ventricular cavity. LV = left ventricle.

All contours were traced twice, using two different tracing methods. Both the systemic RV, and the subpulmonary LV were subjected to both analysis methods. *Method A*: Contour tracing was first performed including the papillary muscles and trabeculations in the ventricular cavity, by tracing immediately within the apparently compact layer of the myocardium. A continuous movie display of the slice being evaluated was used to enhance differentiation between trabeculations, papillary muscles and the ventricular free wall. Although the exclusion of complex RV trabeculations and papillary muscles is relatively easy in ED, optimal differentiation is especially important in the ES phases as trabeculations and papillary muscles compress and fold during systole making the end-systolc border of trabeculations and blood volume less distinct. This method resulted in smooth contours (figure 2, method A). *Method B*: Contour tracing was then performed excluding the papillary muscles and trabeculations from the ventricular volume. This was done by tracing around these structures if attached to the ventricular wall and by tracing them separately if not attached to the ventricular wall. This resulted in irregular endocardial contours (figure 2, method B).

To ensure that found differences would only be due to the impact of papillary muscles and trabeculations both analysis methods were performed on the same ED and ES phases, and the same slices for each patient. Duration of RV volume and function analysis was recorded for both analysis methods.

### Statistical methods

For statistical analyses SPSS 12.0.1 (SPSS Inc., Chicago, Illinois) for Windows was used. P values < 0.05 were considered statistically significant. The shift between the two tracing methods was compared with the two-tailed paired *t *test, calculating mean, standard deviation and statistical significance of the differences. The agreement between the two tracing methods was assessed and visualized with the method and plots as described by Bland and Altman. [19] Agreement between RV SV and LV SV for both analysis methods was assessed using a two-tailed paired *t *test.

Intra- and inter-observer reproducibility of the two analysis methods was determined from the mean value and the differences between the two measurements. The coefficient of variability was calculated as the standard deviation of the difference of the paired measurements divided by the mean of the average of the paired measurements, and expressed as a percentage. An extension of the Bland-Altman method was used to assess the statistical significance of differences in intra- and interobserver reproducibility between the two analysis methods. A log transformation of the squared differences between the two measurements was performed. If the squared difference was zero, we replaced the value by the next smallest value multiplied by 0.5. A two-tailed paired *t *test of the logged squared differences was performed thereafter.[16]

## Results

### Differences in measured volumes and function (Additional file 1)

**Figure 3:**
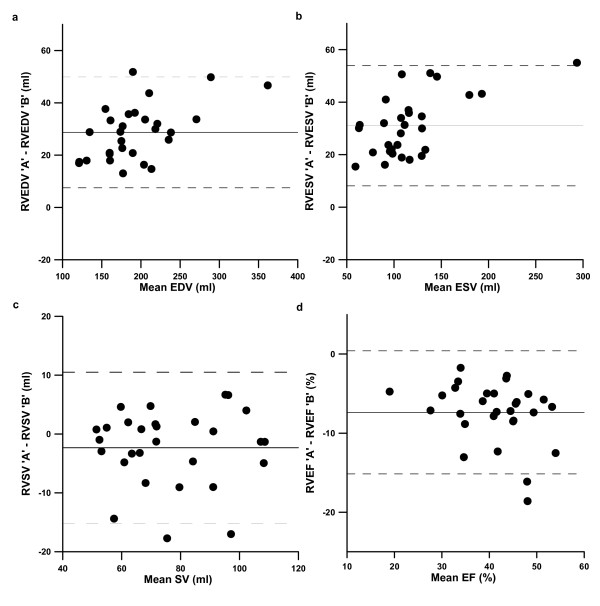
**Bland-Altman plots demonstrating the systematic differences in measured systemic right ventricular volumes and function between Method A and Method B**. Bland-Altman plots demonstrate on the X-axis the mean value of Method A and Method B for each parameter (**a**. mean end diastolic volume; **b**. mean end systolic volume; **c**. mean stroke volume; **d**. mean ejection fraction), and on the Y-axis the difference between the two analysis methods for the same parameter. The solid line represents the mean value of the difference for each method, the dotted lines represent ± 2 SD. RVEDV = right ventricular end diastolic volume; RVEF = right ventricular ejection fraction; RVESV = right ventricular end systolic volume; ml = millilitre; SD = standard deviation; RVSV = right ventricular stroke volume. The 'A' or 'B' behind parameters indicate that values were obtained using Method A or Method B respectively.

Including trabeculations and papillary muscles in the systemic RV volume (Method A) resulted in significantly higher outcome measures for EDV with a mean difference of 28.7 ml (95% CI 24.7 – 32.7, p < 0.001), and for ESV with a mean difference of 31.0 ml (95% CI 26.7 – 35.4, p < 0.001), compared to when structures were excluded from the ventricular volume (Method B). This resulted in a significantly lower calculated systemic RV EF with a mean difference of 7.4% (95% CI -8.9 – -5.9, p < 0.001). No significant changes were found for SV and CO. Bland-Altman plots were used to visualize the systematic differences between Method A and B (figure 3).

We found no statistically significant differences between RV SV and LV SV, when trabeculations and papillary muscles were included in the RV and LV volumes (75.5 ± 18.5 ml vs. 71.1 ± 23.9 ml; p = NS), neither when structures were excluded from the RV and LV volumes (77.8 ± 18.4 ml vs. 69.3 ± 22.3 ml, p = NS).

Analysis time was significantly shorter when using Method A (20 ± 3 min) compared to Method B (26 ± 4 min); p < 0.001.

### Observer reproducibility

Analysis Method A lead to a lower coefficient of variability for all outcome measures compared to Method B. This indicates superior intra- and inter-observer reproducibility when including trabeculations and papillary muscles in the ventricular volume compared to excluding these structures. Although these differences were not found to be statistically significant for the intra-observer measurements, we found statistically significant difference in the inter-observer reproducibility of systemic RV SV (p < 0.05) and EF (P < 0.01), favoring the inclusion of structures in the ventricular volume (Additional file 2).

## Discussion

Including trabeculations and papillary muscles in the systemic RV cavity lead to a substantially higher measured EDV and ESV and a substantially lower calculated EF, compared to excluding these structures from the volume of the cavity. Although the influence of these structures on measured ventricular volumes in individuals with normal cardiac anatomy seems of marginal clinical importance,[16,17] their influence on systemic RV volumes was found to be striking. Moreover, there were statistically significant differences in analysis time and in reproducibility between CMR analysis methods.

Although CMR is considered the most accurate diagnostic tool for the assessment of ventricular volumes and function, [11-13,20,21], there is no consensus in the literature on the role of trabeculations and papillary muscles. In anatomically normal hearts, Lorenz et al. excluded trabeculations and papillary muscles from the LV and the RV cavity,[12] whereas Rominger et al. chose to include these structures.[13] Most authors, however, refrained from specifying the role of trabeculations and/or papillary muscles in their analysis method. [22-25] Similar incompleteness in methodology of CMR analysis is seen in literature regarding patients with congenital heart defects, and with systemic RVs in specific. Although Helbing et al. excluded papillary muscles and the moderator band from the RV cavity of patients with congenital heart defects,[10] and Lidegram et al. chose to include both structures in the cavity of the systemic RV,[26] most authors are less specific on the role of these structures in their analysis method.[11,21,27]

The influence of trabeculations and papillary muscles on LV and RV measurements in healthy subjects and patients with known cardiac disease (patients with congenital heart defects were excluded) has been studied previously, using similar analysis protocols as were used in our study.[16,17] In anatomically normal hearts, both Sievers et al. and Papavassiliu et al. found significant differences in measured left and right ventricular volumes and function when comparing both CMR analysis protocols. However, both authors concluded that the observed differences in ventricular volumes and function were too small to influence clinical decision making, and advised the inclusion of all structures in the ventricular cavity. Including structures not only shortened analysis time, Papavassiliu et al. also demonstrated superior reproducibility for several outcome measures when using this analysis protocol.[16,17] Our observations in systemic RVs differ from those of Sievers and Papavassiliu, as we found systematic and large differences in measured systemic RV volumes and function between the two analysis methods. This study indicates the importance of a consistent approach to cavity delineation relative to the trabeculations and papillary muscles, to avoid misinterpretation of measurements and erroneous clinical decision making.[5,6]

Although the true values of systemic RV volumes and function remain unknown, and in spite of tricuspid valve regurgitation in some patients, we found no significant differences between the measured RV and LV SV by either method of analysis. However, delineation of the RV cavity boundary outside the trabeculations and papillary muscles had the advantages of shorter analysis time and better inter-observer reproducibility. We therefore recommend the use of this approach in routine CMR measurements of systemic RV volumes, at least when comparable systems for CMR acquisition and volume analysis are being used.

### Study limitations

The sample size of 29 patients in two distinct clinical categories is relatively small. The methods used were not suitable for determining which of the analysis approaches measured systemic RV volumes more accurately. Measurements of systemic RV mass were not attempted, and remain challenging given the relative amount of trabeculated RV myocardium. The slice thickness of 8 mm may not have been optimal for clear delineation of the trabeculations, making the definition of the boundaries between trabeculations and blood, and between trabeculations and apparently compact myocardium hard to define in some cases. Moreover, as myocardial boundaries were first defined at end diastole, detecting the corresponding boundary at end systole could be difficult, due to elimination of blood from the inter-trabecular spaces at end systole. The important issue of inter-study reproducibility was not addressed by this study. Between studies, volume measurements might be affected by variation in the relative positioning of the basal short axis slice, and by variables such as the shimming of the magnet and the reliability of ECG triggering.

## Conclusion

We found the method of systemic RV cavity delineation to affect the measurements of cavity volume, given the CMR acquisition and analysis systems used. We recommend cavity delineation inside the apparently compact myocardium of the RV but outside the trabeculations and papillary muscles for routine clinical measurements of systemic RV volumes as this approach took less time and gave more reproducible values.

## Abbreviations

ccTGA: congenitally corrected transposition of the great arteries; CHD: congenital heart defect; CMR: cardiovascular magnetic resonance; CO: cardiac output; EDV: end diastolic volume; EF: ejection fraction; ESV: end systolic volume; LV: left ventricle; RV: right ventricle; SV: stroke volume; TGA: transposition of the great arteries

## Competing interests

The authors declare that they have no competing interests.

## Authors' contributions

MW designed the study format, carried out the literature search, the data collection, and analysis, the statistical analysis and the interpretation of data, carried out the manuscript writing, and gave final approval. FB designed the study format, carried out the literature search, the data collection, and analysis, the statistical analysis and the interpretation of data, carried out the manuscript writing, and gave final approval. MG substantially contributed to the conception and design of the study, performed data acquisition, critically revised the article, and gave final approval. BB substantially contributed to the conception and design of the study, the analysis of data, critically revised the article, and gave final approval. AD substantially contributed to the conception and design of the study, the analysis of data, critically revised the article, and gave final approval. WH substantially contributed to the conception and design of the study, the analysis of data, critically revised the article, and gave final approval. JT substantially contributed to the conception and design of the study, the statistical analysis of data, critically revised the article, and gave final approval. BM substantially contributed to the conception and design of the study, the analysis of data, the interpretation of data, critically revised the article, and gave final approval.

## Supplementary Material

Additional file 1 Measurements of systemic right ventricular volumes and function by mean of CMR. Click here for file

Additional file 2 Intra- and inter-observer reproducibility of measurements. Click here for file
